# On the Assimilation of Milk in the Intestines of the New-born

**Published:** 1880-01

**Authors:** 


### PHYSIOLOGY.

ON THE ASSIMILATION OF MILK IN THE INTESTINES OF THE NEW BORN. By Forster. *Mitt d. Gesellschf. Morph zu. München*, March 6, 1879. *Revue des Sc. Méd.*, July, 1879.

These researches were made on a child four months old. The child took from 1,000 to 1,200 grams of a mixture of four parts of cow's milk and one part of thin rice water per day. Its weight increased about 150 grams a week. For eleven consecutive days the food eaten and the discharges were accurately weighed. During this time the child ingested 13.04 liters of the mixture, that is, 1.218 liters a day; the solid contents of which was 136.08 grams. These solids were utilized in the intestines of the child in the proportion of 6.35 per cent., that is to say double the quantity of what is utilized in the adult, but considerably inferior to that which is afforded by flesh. The fat was the element least readily absorbed. The fæces during the milk diet did not contain the least trace of albumen or sugar, but they contained from 30 to 40 per cent. of fat or fatty acids, and 34 per cent. of ash, one-third of which is represented by calcium. Large quantities of soap were found, especially earthy soaps. Of the 87.08 grams of ash in the milk taken during the eleven days, 32.01 were found in the fæces; and of the 13.56 grams of calcium, 13.34 grams were recovered. It may be calculated then that the body of the child retained 30 centigrams of calcium a day, or 2-01 grams a week, which corresponds to 19 grams of bone a week, or nearly a kilogram in a year.

